# Targeted intracellular delivery of dimeric STINGa by two pHLIP peptides for treatment of solid tumors

**DOI:** 10.3389/fphar.2024.1346756

**Published:** 2024-03-01

**Authors:** Anna Moshnikova, Michael DuPont, Marissa Iraca, Craig Klumpp, Hannah Visca, Dana Allababidi, Phoebe Pelzer, Donald M. Engelman, Oleg A. Andreev, Yana K. Reshetnyak

**Affiliations:** ^1^ Physics Department, University of Rhode Island, Kingston, RI, United States; ^2^ Department of Chemical Engineering, University of Rhode Island, Kingston, RI, United States; ^3^ Molecular Biophysics and Biochemistry Department, New Haven, CT, United States

**Keywords:** tumor acidity, cold tumors, biophysics, imaging, immuno-suppressive tumors

## Abstract

**Introduction:** We have developed a delivery approach that uses two pHLIP peptides that collaborate in the targeted intracellular delivery of a single payload, dimeric STINGa (dMSA).

**Methods:** dMSA was conjugated with two pHLIP peptides via S-S cleavable self-immolating linkers to form 2pHLIP-dMSA.

**Results:** Biophysical studies were carried out to confirm pH-triggered interactions of the 2pHLIP-dMSA with membrane lipid bilayers. The kinetics of linker self-immolation and dMSA release, the pharmacokinetics, the binding to plasma proteins, the stability of the agent in plasma, the targeting and resulting cytokine activation in tumors, and the biodistribution of the construct was investigated. This is the first study demonstrating that combining the energy of the membrane-associated folding of two pHLIPs can be utilized to enhance the targeted intracellular delivery of large therapeutic cargo payloads.

**Discussion:** Linking two pHLIPs to the cargo extends blood half-life, and targeted delivery of dimeric STINGa induces tumor eradication and the development of robust anti-cancer immunity.

## 1 Introduction

Immune evasion is a hallmark of cancer. Overcoming that evasion to harness the power of the immune system to attack tumors has become a widely employed strategy (Lesterhuis et al., 2011; Hainaut and Plymoth, 2013). Activation of the stimulator of interferon genes (STING) pathway causes the release of factors that trigger the immune response in the tumor microenvironment (TME) (Woo et al., 2014). As a consequence, a variety of STING agonist (STINGa) therapeutic molecules have been developed and tested in anti-tumor strategies (Klarquist et al., 2014). However, the poor pharmacokinetics of STINGa and nonspecific systemic immuno-activation required intra-tumoral dosing in most cases, significantly limiting applications. In general, the first clinical trials have resulted in disappointingly modest efficacy (Motedayen Aval et al., 2020). Also, recent findings indicate that, under certain conditions, the STING pathway can also be implicated in promoting tumor burdens, which worsens disease outcomes (Decout et al., 2021). These findings led to a realization that kinetics and strength of activation are significant factors for efficacy: transient strong activation leads to immune activation and tumor suppression, while prolonged weak activation leads to further immuno-suppression promoting tumor development. Further, it is now clear that activation of the STING pathway in different types of cells within the TME has different benefits (Decout et al., 2021; Chamma et al., 2022). Reprogramming of M2-type tumor-associated macrophages (M2-TAMs) toward an M1 phenotype, suppression of cancer-associated fibroblasts (CAFs) and myeloid-derived suppressor cells (mMDSCs), as well as activation of dendritic cells (DCs) to train T-cells are advantageous. The activation of T-cells is also associated with the development of pro-apoptotic signals. Thus, a successful STING approach should rely on the targeted delivery of potent STINGa to induce the transient activation of the STING pathway in myeloid and cancer cells. pH Low Insertion Peptides (pHLIP^®^ peptides) triggered by local acidity to fold and directionally insert across the membranes of acidic cells (Reshetnyak et al., 2020) were used for intracellular delivery of therapeutic cargoes (Cheng et al., 2015; Wyatt et al., 2018a; Svoronos et al., 2020; Gayle et al., 2021; Moshnikova et al., 2022a), including targeting of STINGa to cancer cells, M2-TAMs, CAFs, DCs and mMDSCs within TME (Moshnikova et al., 2022b). In this study we used two pHLIP peptides for a targeted delivery of dimeric STINGa for tumor eradication and development of anti-cancer immunity.

## 2 Materials and methods

### 2.1 Synthesis of pHLIP-STINGa compounds

Modified monomeric and dimeric STING agonists mMSA-PAB-SS-Py and Py-SS-Et-dMSA-Et-SS-Py, respectively, were synthesized and purified by Iris Biotech GmbH. pHLIP peptides consisting of all D amino acids were synthesized and purified at CSBio and Biosynth. To prepare pHLIP-mMSA and 2pHLIP-dMSA, Var3 pHLIP peptide was synthesized with single Cys residues at the membrane-inserting end: ADQDNPWRAYLDLLFPTDTLLLDLLWCG. pHLIP peptide was mixed with mMSA-PAB-SS-Py or Py-SS-Et-dMSA-Et-SS-Py in dimethyl sulfoxide (DMSO) at molar ratios of 1:1 or 2:1, respectively. Sodium phosphate buffer (100 mM) containing 150 mM NaCl at pH 7.4 saturated with argon was added to the reaction mix (1/10 of the total volume) and the reaction mixture was kept for 2 h at room temperature (RT). pHLIP-mMSA and 2pHLIP-dMSA constructs were purified by reverse phase high-performance liquid chromatography (HPLC) using Zorbax SB-C18 or Zorbax SB-C8, 9.4 × 250 mm, 5 μm columns (Agilent Technology) with a gradient from 20% to 80% acetonitrile in water containing 0.05% trifluoroacetic acid (TFA). For the preparation of a fluorescent version of 2pHLIP-dMSA (2(ICG-pHLIP)-dMSA), an N-terminal acetylated version of the pHLIP peptide with a lysine introduced near the N-terminus (AKDQDNPWRAYLDLLFPTDTLLLDLLWCG) was used. First, pHLIP was conjugated with Py-SS-Et-dMSA-Et-SS-Py, followed by purification. Then, ICG-NHS ester (Iris Biotech GmbH) was conjugated with the lysine residue at the N-terminal end of pHLIP in DMSO at molar ratio of 2.5:1. Sodium bicarbonate buffer (100 mM) at pH 8.3 was added to the reaction mix (1/10 of the total volume) and the reaction mixture was kept at RT until the conjugation was completed. The final purification was performed as described above. The products were lyophilized and characterized by matrix-assisted laser desorption/ionization-time of flight (MALDI-TOF) mass-spectrometry and analytical HPLC. The molecular weight of pHLIP-mMSA and 2pHLIP-dMSA are 3,781 Da and 7,574 Da, respectively. The concentration of pHLIP-STINGa conjugates was determined by absorbance using the following molar extinction coefficients: for pHLIP-mMSA *ε*_325_ = 15,000 M^−1^cm^−1^; *ε*_325_ = 30,000 M^−1^cm^−1^ for 2pHLIP-dMSA and *ε*_800_ = 137,000 M^−1^cm^−1^ for 2(ICG-pHLIP)-dMSA.

### 2.2 Biophysical studies

The interactions of pHLIP-mMSA and 2pHLIP-dMSA with liposomes were investigated by recording the construct’s fluorescence and circular dichroism (CD) using a PC1 spectrofluorometer (ISS) and a MOS-450 spectrometer (Bio-Logic Science Instruments), respectively, with temperature control set to 25°C. Liposomes, constituting of large unilamellar vesicles were prepared by extrusion. 1-palmitoyl-2-oleoyl-*sn*-glycero-3-phosphocholine (POPC) lipids (Avanti Polar Lipids) in chloroform were desolvated on a rotary evaporator and dried under vacuum for a minimum of 2 h. The phospholipid film was rehydrated in 2 mM citrate phosphate buffer, pH 7.4, vortexed, and passed through the extruder (using a 50 nm membrane pore size) 21 times.

Fluorescence spectra were recorded from 310 nm to 390 nm at an excitation wavelength of 295 nm and 1.0 mm sized slits. The excitation polarizer was set to 54.7° (“magic angle”) while the emission polarizer was set to 0° in order to reduce Wood’s anomalies. CD spectra were recorded from 190 to 260 nm with a step size of 1 nm. The concentrations of pHLIP-mMSA/2pHLIP-dMSA and POPC were 7 μM and 1.4 mM, respectively.

The pH-dependent insertion of pHLIP-mMSA or 2pHLIP-dMSA into the lipid bilayer of POPC liposomes was studied by monitoring changes in the molar ellipticity as a function of pH. After the addition of aliquots of citric acid, the pHs of solutions containing pHLIP-mMSA/2pHLIP-dMSA and POPC liposomes were measured using an Orion PerHecT ROSS Combination pH Micro Electrode and an Orion Dual Star pH and ISE Benchtop Meter. The millidegree ellipticity values were plotted as a function of pH. The pH-dependence was fit with the Henderson-Hasselbach equation to determine the cooperativity (*n*) and the mid-point ( pK ) of transition. The fitting equation used was the following:

$$pH\;dependence=S2+\frac{S3-S2}{1+10^{n\left(pH-\text{pK}\right)}}\text{ for }a\text{ single transition}$$

where *S2* represents CD signal of pHLIP-mMSA or 2pHLIP-dMSA in presence of POPC at pH 7.4, when pHLIP peptides are adsorbed, but not inserted across lipid bilayer, and *S3* represents CD signal of pHLIP-mMSA or 2pHLIP-dMSA in presence of POPC at pH 4.5, when pHLIP peptides are inserted across lipid bilayer of membrane.

Fluorescence kinetics was measured using a SFM-300 mixing system (Bio-Logic Science Instruments) in combination with the MOS-450 spectrometer with the temperature control set to 25°C. All samples were degassed before measurement to minimize air bubbles in the samples. pHLIP-mMSA/2pHLIP-dMSA and POPC samples were incubated overnight to reach equilibrium, when most of the agent was associated with liposome lipid bilayers. To follow insertion into a membrane, a solution containing 14 μM pHLIP-mMSA/2pHLIP-dMSA and 2.8 mM POPC was mixed with citric acid to lower the pH from pH 7.4 to 4. To follow exit of pHLIP-mMSA/2pHLIP-dMSA from the membrane, a low pH solution containing 14 μM pHLIP-mMSA/2pHLIP-dMSA and 2.8 mM POPC was mixed with dibasic phosphate buffer to raise the pH from 4 to 7.4. To monitor the fluorescence intensity changes during pHLIP-mMSA/2pHLIP-dMSA insertion induced by the pH change, the emission signal was observed through a cut-off 320 nm filter at an excitation of 295 nm.

All data were fit to the appropriate equations by nonlinear least squares curve fitting procedures employing the Levenberg Marquardt algorithm using Origin 8.5.

### 2.3 Binding of mMSA and dMSA to STING protein

Binding of mMSA and dMSA to STING protein was measured with human STING WT binding kit (Cisbio). Briefly: mMSA (MSA2, from MCE), dMSA (Iris Biotech GmbH) or 2′3′-cGAMP (Cisbio) control were added in increasing concentrations to the 96-well microplate (PerkinElmer), followed by addition of 6His-tagged human STING WT protein. STING WT ligand d2 reagent was premixed with 6His Tb antibodies, and it was added to each well and the plate was incubated for 3 h at RT. Fluorescence intensities at 616 nm and 665 nm excited at 450 nm were recorded on the SpectraMax ID5 plate reader (Molecular Devices). The homogeneous time resolved fluorescence (HTRF) ratio was calculated to establish the binding of each agent to the STING protein and to calculate EC50.

### 2.4 Binding to human serum albumin

Binding affinities of pHLIP-mMSA and 2pHLIP-dMSA to human serum albumin (HSA) (MilliporeSigma) were investigated using a series of competitive fluorescence binding assays measured on a SpectraMax ID5 plate reader with SoftMax 7.1.2 software. HSA was initially dissolved to 500 μM in deionized water and further diluted using pH 7.6 phosphate buffer. Dansylamide (DNSA) or dansylglycine (DNSG) (both from Tokyo Chemical Industry) were used as fluorescent markers, which are known to bind to specific sites on HSA. Each well contained 100 μL of sample consisting of the fluorescent marker DNSA or DNSG (1 μM), with different concentrations of pHLIP agents (0, 5, or 10 μM) and concentrations of HSA ranging from 0 μM to 100 μM. Samples were incubated for 2 h at 37°C. As controls, warfarin and ibuprofen, drugs known to bind different sites on HSA, were used instead of pHLIP agents. Fluorescence measurements from each well were performed at 485 nm with excitation at 365 nm at 37°C. As a control, binding of DNSA and DNSG were probed with each agent in absence of HSA. Before the measurements a plate reader optimization test was run. Data were fitted individually and globally using the Michaelis-Menten equation. In the global fits, a common Vmax value was used, established for 0 μM of pHLIP agents for both DNSA and DNSG. Data was fitted by nonlinear least squares curve fitting procedures employing the Levenberg Marquardt algorithm using Origin 8.5.

### 2.5 Self-immolation kinetics

To trigger S-S cleavage and self-immolation of the linker connecting pHLIP peptide and mMSA or dMSA payloads, solutions of pHLIP-mMSA or 2pHLIP-dMSA were treated with dithiothreitol (DTT). At different time points (from 30 min to 48 h of treatment) the samples were analyzed by HPLC using a Zorbax SB-C18 (for pHLIP-mMSA) or a Zorbax SB-C8 (for 2pHLIP-dMSA) 4.6 × 250 mm, 5 μm columns with a gradient from 20% to 80% acetonitrile in water containing 0.05% TFA. The chromatograms were recorded at 220 nm, 280 nm and 325 nm. mMSA, dMSA and pHLIP were used as controls at the same HPLC conditions.

### 2.6 Stability in mouse and human plasma

To establish the stability of agents (2pHLIP-dMSA, pHLIP-mMSA, dMSA, mMSA, and pHLIP) in plasma, each agent was mixed with single donor human or BALB/c mouse plasma (Innovative Research) at a concentration of 200 μM, and kept in plasma for 0, 1, 2, 4 or 24 h at 37°C. Plasma proteins were precipitated by methanol (1:5 volume ratio of plasma to methanol) and centrifuged for 10 min at 13.4 rpm. The supernatant was collected and analyzed by HPLC using a Zorbax SB-C18 4.6 × 250 mm, 5 μm column with a gradient from 20% to 80% acetonitrile in water containing 0.05% TFA. Chromatograms were recorded at 220 nm, 280 nm and 325 nm.

### 2.7 Activation of IFN in M2 macrophages

THP-1-Blue™-ISG cells (Invivogen) expressing an interferon (IFN) regulatory factor (IRF)-inducible secreted embryonic alkaline phosphatase (SEAP) reporter construct were used. Cells were maintained in RPMI growth medium supplemented with L-glutamine, sodium pyruvate, 10% fetal bovine serum (FBS), normocin and ciprofloxacin hydrochloride in a humidified atmosphere of 5% CO_2_ and 95% air at 37°C. Cells were seeded in 96-well plates at a density of 75,000 cells/well. To generate M2 polarized macrophages, cells were treated first with 185 ng/mL phorbol 12-myristate 13-acetate (PMA) for 6 h and then 20 ng/mL of interleukin 4 (IL-4) and 20 ng/mL of interleukin 13 (IL-13) (both from PeproTech) were added for another 16 h of treatment. At the completion of polarization, the growth medium was replaced with Dulbecco’s Modified Eagle Medium (DMEM) medium without FBS, pH 6.9, containing increasing amounts of 2pHLIP-dMSA or dMSA (up to 10.0 µM). After a 2-h incubation, an equal volume of RPMI supplemented with 20% heat-inactivated FBS was added, and cells were incubated for another 48 h. SEAP activity was accessed using the QUANTI-Blue™ Solution (Invivogen) to evaluate type I interferon protein levels: 150 μL of the colorimetric reagent was added to 50 μL of cell supernatant for 30 min, 37°C, followed by absorption measurement at 655 nm.

### 2.8 Treatment of mice

All animal studies were conducted at the University of Rhode Island (URI) according to the approved by URI Institution Animal Care and Use Committee (IACUC) animal protocols AN04-12-011 and AN 1920-003. The studies complied with the principles and procedures outlined by the National Institutes of Health for the care and use of animals.

For the treatment of CT26 tumors, 5 × 10^4^ CT26 murine colorectal cancer cells (ATCC, CRL-2638) were injected subcutaneously (SQ) in 100 μL of growth medium into the right flank of female Balb/c mice ranging in age from 7 to 9 weeks (Envigo RMS, Inc.). On day 1, when tumors reached a volume of 100–130 mm^3^, mice were randomized into groups, body weight was measured and agents, including 2pHLIP-dMSA, pHLIP-mMSA, dMSA, mMSA or vehicle, were given as the first intraperitoneal (IP) injection. The second IP injection was given after 48 h mMSA, dMSA and pHLIP-mMSA were given as two IP injections of 300 μM 500 µL. 2pHLIP-dMSA was given as two IP injections of 300 μM 500 μL or 100 μM 500 μL (low dose). The compounds were dissolved in DMSO as a stock solution and transferred to PBS (vehicle). The residual DMSO in the final solution injected into animals was less than 2%. Tumor volume and body weight were measured 3 times per week throughout the study. Measurements of tumors were performed using calipers, and the tumor volume (*V*) was calculated with the formula:

$$V=0.52∙L∙W^{2}$$

where *L* is the length and *W* is the width of the measured tumor. Mice were removed from the study and euthanized when the tumor volume was greater than 2,000 mm^3^.

In the 2pHLIP-dMSA treated groups the mice that stayed tumor-free were re-challenged with tumor cells injected into the tumor-free flank on day 65 after the first injection of 2pHLIP-dMSA. Tumor-free mice were kept for an additional 40 days (total of 105 days after the treatment with 2pHLIP-dMSA) and then were euthanized.

### 2.9 Biodistribution and PK

For biodistribution studies 5 × 10^4^ of CT26 cancer cells were injected SQ in 100 µL of growth medium into the right flank of female Balb/c mice and tumors were grown until they reached 150–250 mm^3^ in volume. Then a single retro-orbital (RO) injection of 50 μM 100 μL of 2(ICG-pHLIP)-dMSA was performed. Animals were euthanized at 5 min, 1, 4, 24, 48, 72, and 120 h post-injection, blood was collected in K_2_ EDTA vacutainer blood collection tubes (BD), and necropsy was performed immediately after animals were euthanized. Blood, tumors and major organs (kidney, liver, spleen, pancreas, lung, heart, large and small intestines, bone, muscle, brain) were collected, and imaged *ex vivo* immediately after necropsy. Blood (200 µL) was imaged in 96-well plates with black bottom and walls. The zero-time point (0 min) was obtained by imaging of 2(ICG-pHLIP)-dMSA diluted in blood collected from a control mouse that did not receive any injection (the dilution was made based on the assumption that a mouse contains 80 mL/kg of blood).

The *in vivo* and *ex vivo* bright field and near-infrared fluorescent imaging employed a Stryker 1588 AIM endoscopic system with L10 AIM Light Source (808 nm excitation and collection of light in the range of approximately of 815–850 nm), and a 1588 AIM Camera using a 10 mm scope. The lens was kept at a fixed distance from the surface of the organs, within an enclosed (light-protected) area. Calibration curves converting laser and gain settings were established by imaging of pHLIP-ICG solution at different concentrations. The *ex vivo* fluorescence imaging of tissue specimens was performed using three different laser settings and six different gain settings. The digital fluorescence images were split into RGB channels, with the green channel being analyzed using ImageJ program to calculate the average fluorescence intensity. All average intensities were adjusted based on their capture settings and using calibration curves to present fluorescence intensities corresponding to the unified (the same) settings.

### 2.10 ELISA on blood and tumor samples

To establish levels of cytokines in blood and tumor samples, 5 × 10^4^ CT26 cancer cells were injected SQ in 100 µL of growth medium into the right flank of female Balb/c mice. When tumors reached 250–500 mm^3^ in volume, the mice received a single IP injection of 300 μM 500 µL 2pHLIP-dMSA or no injections. Animals were euthanized at 6- and 24-h post-injection, blood and tumors were collected. Blood samples were kept for 40 min at RT, centrifuged at 5,000 *g* for 20 min at +4°C and supernatant (serum) was collected. Tumors were frozen in liquid nitrogen. Both the serum and the tumor tissue samples were kept at −80°C until further processing and analysis. The tumor samples were processed while on ice, using a bullet blender (Next Advance) with 1 mm diameter zirconium silicate beads (Next Advance). The supernatant of the processed tumors was used for enzyme-linked immunoassay (ELISA) assays. Matched antibody pair kits for mouse TNF alpha (Sino Biological), mouse IL-6 (Abcam), mouse IFN gamma (Invitrogen), mouse IFN beta (Bio-Techne), and a precoated mouse IFN alpha kit (Invitrogen) were used. For TNF alpha and IFN beta the capture antibodies were diluted in PBS (Cytiva), while the IL-6 and IFN gamma capture antibodies were diluted in their respective coating buffers. The diluted capture antibodies were incubated in the plates overnight at +4°C and washed the next day with PBS/Tween washing buffer (Sigma-Aldrich). The TNF alpha plate was blocked using 2% BSA (Thermo-Scientific) diluted in washing buffer, the IFN beta plate was blocked using 1% BSA diluted in PBS, while the IFN alpha, IFN gamma, and IL-6 plates were blocked using their respective dilution buffers. Blocking was done for 2 h at RT on an orbital shaker at 200 rpm. After blocking, the plates were washed and then incubated with the diluted tumor and serum samples along with the corresponding standard solutions for each ELISA kit. The samples were incubated for 2 h at RT on an orbital shaker at 200 rpm. The TNF alpha plate was incubated for 1 h at RT with a diluted detection antibody conjugated with horseradish peroxidase (HRP). The IL-6, IFN alpha, IFN beta, and IFN gamma plates were incubated for 1 h at RT with a diluted detection antibody conjugated with biotin followed by incubation for 1 h at RT with their respective diluted HRP-streptavidin conjugates. All plates were washed and incubated with 3,3′,5,5′-tetramethylbenzidine (TMB) (Invitrogen) and peroxide solution mixed at a ratio of 1:1 (Thermo Scientific) for up to 20 min, then a stop solution (10% H_2_SO_4_) was added to the plates. The signal from the wells was quantified by absorbance measured at 450 nm using a Bio-Rad iMark microplate reader. Antibody standards were used to plot calibration curves.

### 2.11 Statistical analysis

The Kolmogorov-Smirnov two-tailed nonparametric test was used to establish *p-levels*.

## 3 Results

### 3.1 Binding affinity of monomeric and dimeric STINGa

The majority of synthetic STINGa, used for systemic administration, are monomers like MSA-2 (mMSA), which occupy two adjacent binding sites in the STING protein dimer. Perceiving that linking two STINGa molecules might enhance affinity via entropic effects, several dimers were designed based on the MSA-2 monomer (dMSA) (Pan et al., 2020). Indeed, dimers bound more strongly to the STING protein (Pan et al., 2020). We confirmed this finding by testing the binding of both mMSA and dMSA to the STING protein and establishing that binding affinities were 7.5 µM for mMSA and 24 nM for dMSA, so the dimer is much more potent than the monomer (Supplementary Figure S1). Despite their high protein potency, dimers exhibit limited membrane permeability and significantly reduced cellular activity. Their potential bioavailability is low compared with MSA-2, which was developed as an oral drug. In any case, untargeted delivery of STINGa leads to systemic activation of the immune system and is associated with systemic toxicity.

### 3.2 Chemical conjugation with pHLIP

**FIGURE 1:**
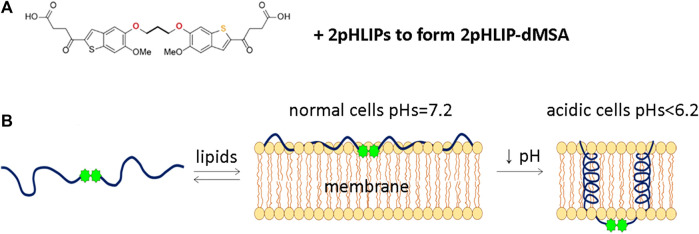
Schematic representation of 2pHLIP-dMSA interaction with a membrane lipid bilayer. The chemical structure of dMSA and proposed interaction with 2 pHLIP peptides are shown **(A)**. In **(B)**, schematics are shown of 2pHLIP-dMSA reversible interactions with the membranes of cells with normal surface pH (pH = 7.2) in healthy tissue and membranes of acidic cells with surface pH < 6.2 found in diseased tissues.

We have developed a delivery approach that uses multiple pHLIP peptides that collaborate in the targeted intracellular delivery of a single payload. Both mMSA and dMSA payloads were modified with the attachment of S-S cleavable self-immolating linkers to their carboxyl groups. A disulfide bond was exchanged with the SH group of a unique Cys residue placed at the C-terminal end of a version of a pHLIP peptide to obtain two compounds. In 2pHLIP-dMSA two pHLIP peptides were linked to a single dMSA dimeric STINGa (Figure 1A). We would like to note that it is challenging to conjugate and purify a single pHLIP linked to dMSA, since dMSA carries two identical linkers and it is impossible to stop conjugation reaction, since it continues during the purification. In pHLIP-mMSA a single pHLIP peptide was linked with a single mMSA monomeric STINGa (Supplementary Figure S2A). We used a Var3 version of pHLIP peptide made from D amino acids. In the case of 2pHLIP-dMSA, the energy of folding of two pHLIP peptides is expected to be used in a cooperative manner for the intracellular delivery of the dMSA payload across the membranes of acidic cells (Figure 1B), while in the case of pHLIP-mMSA, a single pHLIP peptide can translocate a single mMSA payload across the membrane of acidic cells (Supplementary Figure S2B).

### 3.3 Kinetics of self-immolation

To confirm that the dMSA payload can be released after the cleavage of the S-S bond, we monitored the kinetics of self-immolation by HPLC analysis as a function of time after the addition of DTT into solutions containing 2pHLIP-dMSA. The cleavage of S-S bonds is followed by the formation of multiple intermediates during the self-immolation process, which is mostly completed within 48 h with the release of dMSA in its original form (Supplementary Figure S3). A similar process, but with a smaller number of intermediates, was observed for self-immolation and release of mMSA, which was completed within 24 h (Supplementary Figure S4).

### 3.4 Biophysical characterization

**FIGURE 2:**
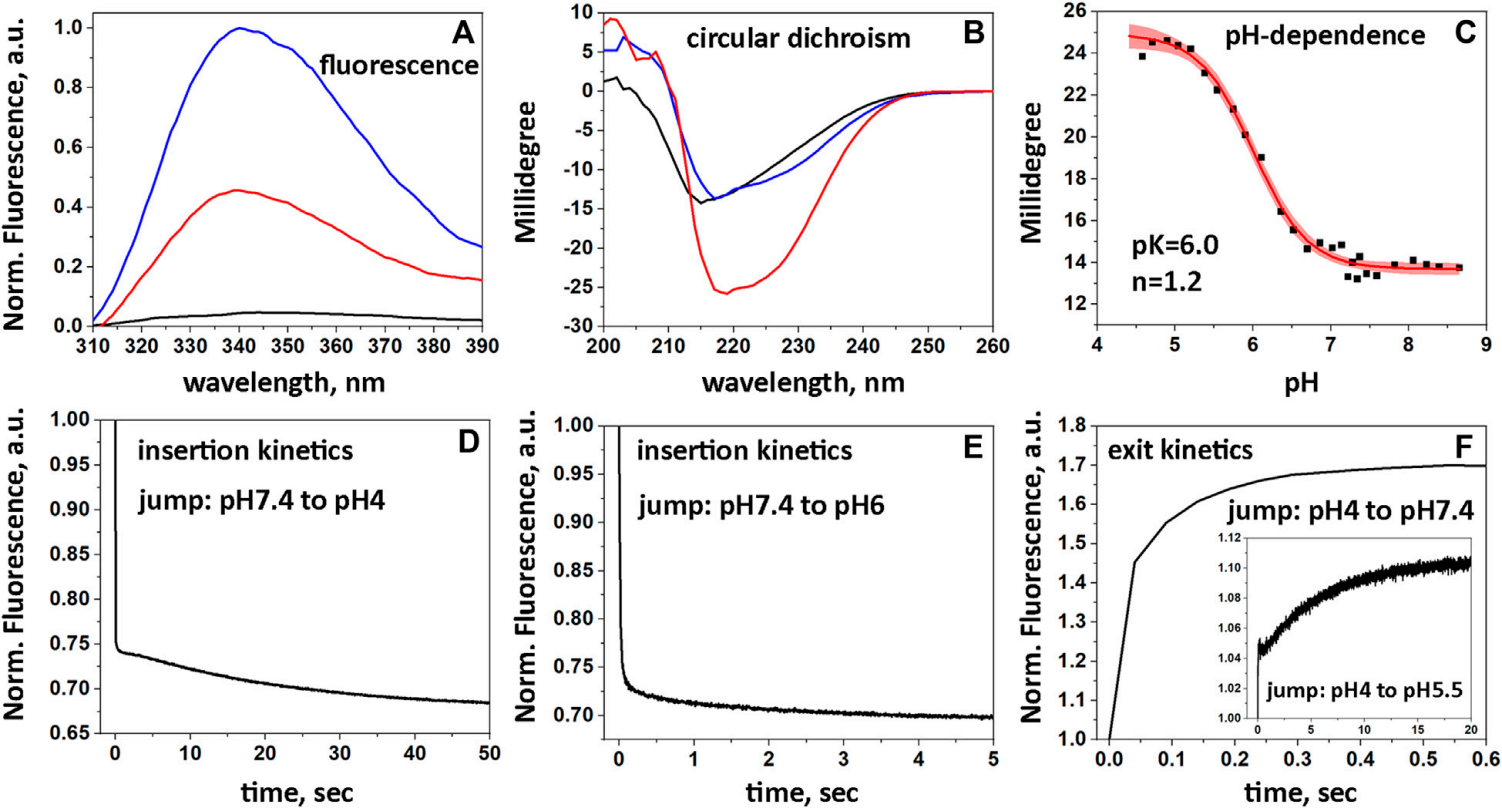
Interactions of 2pHLIP-dMSA with liposome lipid bilayers. Fluorescence **(A)** and CD **(B)** spectra of 2pHLIP-dMSA in solution at pH 7.4 (black lines), in the presence of POPC liposomes at pH 7.4 (blue lines) and in the presence of POPC liposomes at pH 3.5 (red lines) are shown. CD spectral signals were normalized per peptide and multiplied by (−1), since the D-amino acid pHLIP peptides used in the study invert the usual CD spectrum. pH-dependent transitions monitored by changes of CD spectral signal in presence of POPC liposomes (experimental points and fitting curves, red, with 95% confidence interval, pink) are presented **(C)**. Kinetics of fluorescence changes triggered by pH drops **(D, E)** and pH increases **(F)** in presence of POPC liposomes are shown.

Biophysical studies using POPC liposomes confirmed the pH-dependent interactions of 2pHLIP-dMSA with the membrane lipid bilayer. Fluorescence (Figure 2A) and circular dichroism (Figure 2B) measurements reflect the partitioning of pHLIP into the lipid bilayer accompanied by the formation of helical structure in the membrane at low pH. 2pHLIP-dMSA is observed to insert into the POPC bilayer with a pK of 6.0 and a cooperativity of 1.2 (Figure 2C). Interactions of 2pHLIP-dMSA with bilayers were similar to those of pHLIP-mMSA (Supplementary Figures S5A–C). Insertion of 2pHLIP-dMSA into the lipid bilayer triggered by a pH drop from pH 7.4 to 4 was fast (completed within 100 msec) and small conformational adjustments were observed for another 50 s (Figure 2D). The kinetics of membrane insertion of 2pHLIP-dMSA resulting from a pH drop from pH 7.4 to 6 (Figure 2E), which is more similar to biological conditions, was more efficient and faster than the insertion of pHLIP-mMSA (Supplementary Figure S5E). We also followed the exit of the agents from the lipid bilayer induced by a pH rise from pH 4 to 7.4 and from pH 4 to 5.5 (Figure 2F; Supplementary Figure S5F). The exit of 2pHLIP-dMSA is slightly slower, which is better illustrated during the intermediate pH jump (insert in Figure 2F), compared to the exit of pHLIP-mMSA (Supplementary Figure S5F). The data indicates that two pHLIP peptides trigger efficient partitioning of dMSA into a lipid bilayer as the result of a pH drop, with a less efficient exit compared to a single pHLIP-cargo conjugate.

### 3.5 Cellular activation of STING pathway

**FIGURE 3:**
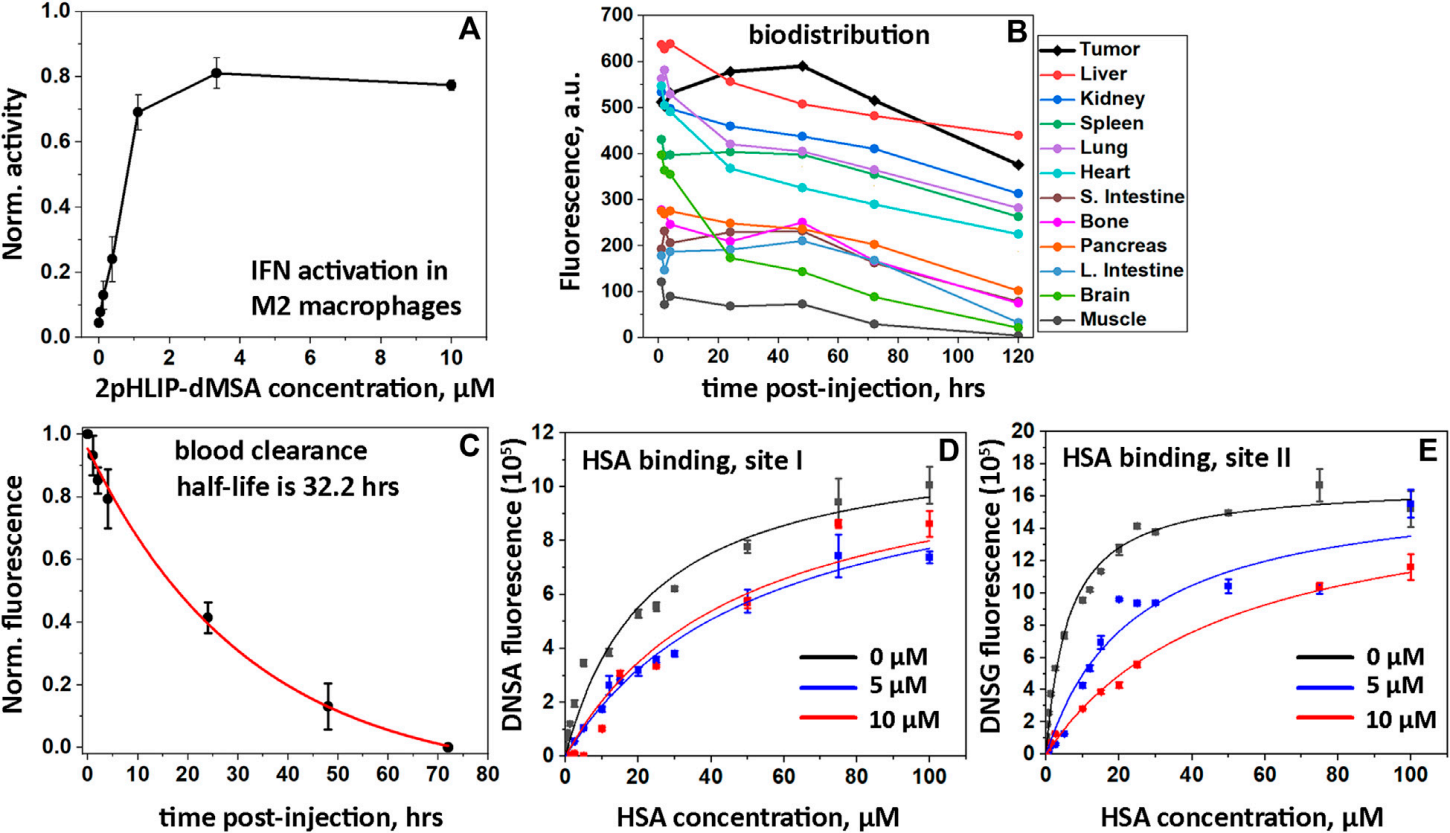
*In vitro* IFN activations, biodistribution, PK and HSA binding. Activation of the IFN signaling pathway induced by 2pHLIP-dMSA in THP1-Blue-ISG cells polarized by PMA, IL-4/IL-13 into M2-type macrophages is shown **(A)**. The results were normalized to the activity of dMSA alone at the maximum concentration tested, which was taken as 1. Kinetics of 2(ICG-pHLIP)-dMSA targeting CT26 tumor and clearance of the agent from major organs are shown **(B)**. The mean fluorescence per area was calculated for each organ and tissue collected at different timepoints after single RO injection of 2(ICG-pHLIP)-dMSA (50 μM 100 μL). Normalized fluorescence recorded in blood, which was collected at different time points after single RO injection of 2(ICG-pHLIP)-dMSA is shown (mean and Sd.) **(C)**. The data were fitted with an exponential function (red line). Changes of DNSA **(D)** and DNSG **(E)** fluorescence with increasing HSA concentration in absence (black symbols) or presence of 5 µM (blue symbols) or 10 µM (red symbols) of 2pHLIP-dMSA. The global fitting was performed with each set of data **(D and E)** and the fitting curves are shown as black, blue and red lines.

To complete the characterization of 2pHLIP-dMSA, the activation of the interferon signaling pathway was confirmed in polarized M2 macrophages. Activation was studied in THP1-Blue cells derived from a human THP-1 monocyte cell line by stable integration of an interferon regulatory factor (IRF)-inducible secreted embryonic alkaline phosphatase (SEAP) reporter construct. We found that THP1-Blue cells exhibited a concentration-dependent activation of IRF signaling when polarized by phorbol 12-myristate 13-acetate (PMA), interleukin 4 (IL-4) and interleukin 13 (IL-13) into M2-type macrophages and treated with 2pHLIP-dMSA (Figure 3A). EC50 for 2pHLIP-dMSA was calculated to be 200 nM, while EC50 for mMSA was established to be 2,200 nM (data not shown). Thus, we confirmed successful intracellular delivery and cytoplasmic release of dMSA by pHLIP peptides and showed that the dimeric construct is significantly more active.

### 3.6 Biodistribution and PK

Biodistribution, tumor uptake and pharmacokinetics (PK) studies were conducted by conjugating a near infrared indocyanine green (ICG) fluorescent dye to the non-inserting end of pHLIP peptides to make a 2(ICG-pHLIP)-dMSA fluorescent construct. When CT26 tumors, established in the right flanks of female Balb/c mice, reached volumes of about 150–250 mm^3^, 2(ICG-pHLIP)-dMSA (50 μM 100 μL) was given as a single retro-orbital (RO) injection. For biodistribution and PK studies, the animals were euthanized, followed by harvesting of blood, tumors and major organs. Fluorescence of the samples was quantitatively measured at 1-, 4-, 24-, 48-, 72-, and 120-h time points after a single RO injection of 2(ICG-pHLIP)-dMSA (Supplementary Figures S6, S7). The kinetics of the fluorescence signal of 2(ICG-pHLIP)-dMSA indicate a significant accumulation of the agent in the tumor from 1 h and up to 48 h followed by a slight decrease of the signal at later time points (Figure 3B). The organ and tissue clearances were slow (Figure 3B). It is important to note that 1) CT26 tumors increase in size over 5 days (120 h time point), so normalized fluorescence may be lower in tumors at later time points; 2) the presence of the agent in an organ does not mean cellular delivery of dMSA, which only occurs in a low pH environment where pHLIP inserts into cell membranes, followed by dMSA cleavage from pHLIP, linker self-immolation, and release of dMSA; and 3) the agent has two ICG dyes, which potentially can affect the clearance profile by significant binding to plasma proteins. Slow tissue clearance is associated with slow blood clearance. Whole blood imaging was performed to establish clearance of the agent (Figure 3C). The observed half-life of 2(ICG-pHLIP)-dMSA is 32.2 h, which is significantly longer compared to any previously investigated pHLIP-cargo conjugate (Gayle et al., 2021; Moshnikova et al., 2022b). We studied binding of 2pHLIP-dMSA with human serum albumin (HSA) in a competitive binding assay using DNSA and DNSG fluorescent dyes, which are known to bind site I and site II on HSA, respectively (Sudlow et al., 1975). Interactions of DNSA or DNSG with increasing concentration of HSA were investigated in the absence and presence of 2pHLIP-dMSA, pHLIP-mMSA, pHLIP, warfarin (binds site I) and ibuprofen (binds site II) (Figures 3D,E; Supplementary Figure S8). The binding of DNSA to HSA was reduced about 2-fold in the presence of 2pHLIP-dMSA, while warfarin at concentration of 10 µM reduced DNSA binding to HSA by 5.3-fold (Supplementary Table S1). The binding of DNSG to HSA was reduced 7.8-fold in the presence of 10 µM of 2pHLIP-dMSA, while ibuprofen reduced DNSG binding to HSA by 5.3-fold (Supplementary Table S1). The obtained results indicate strong binding of 2pHLIP-dMSA to HSA, especially to its site II, which is known to interact with cyclic compounds including ibuprofen, tryptophan, and compounds with free carboxyl groups (Sudlow et al., 1976). We also cannot exclude binding of 2pHLIP-dMSA to other plasma proteins.

### 3.7 Selective activation of cytokines in tumor

**FIGURE 4:**
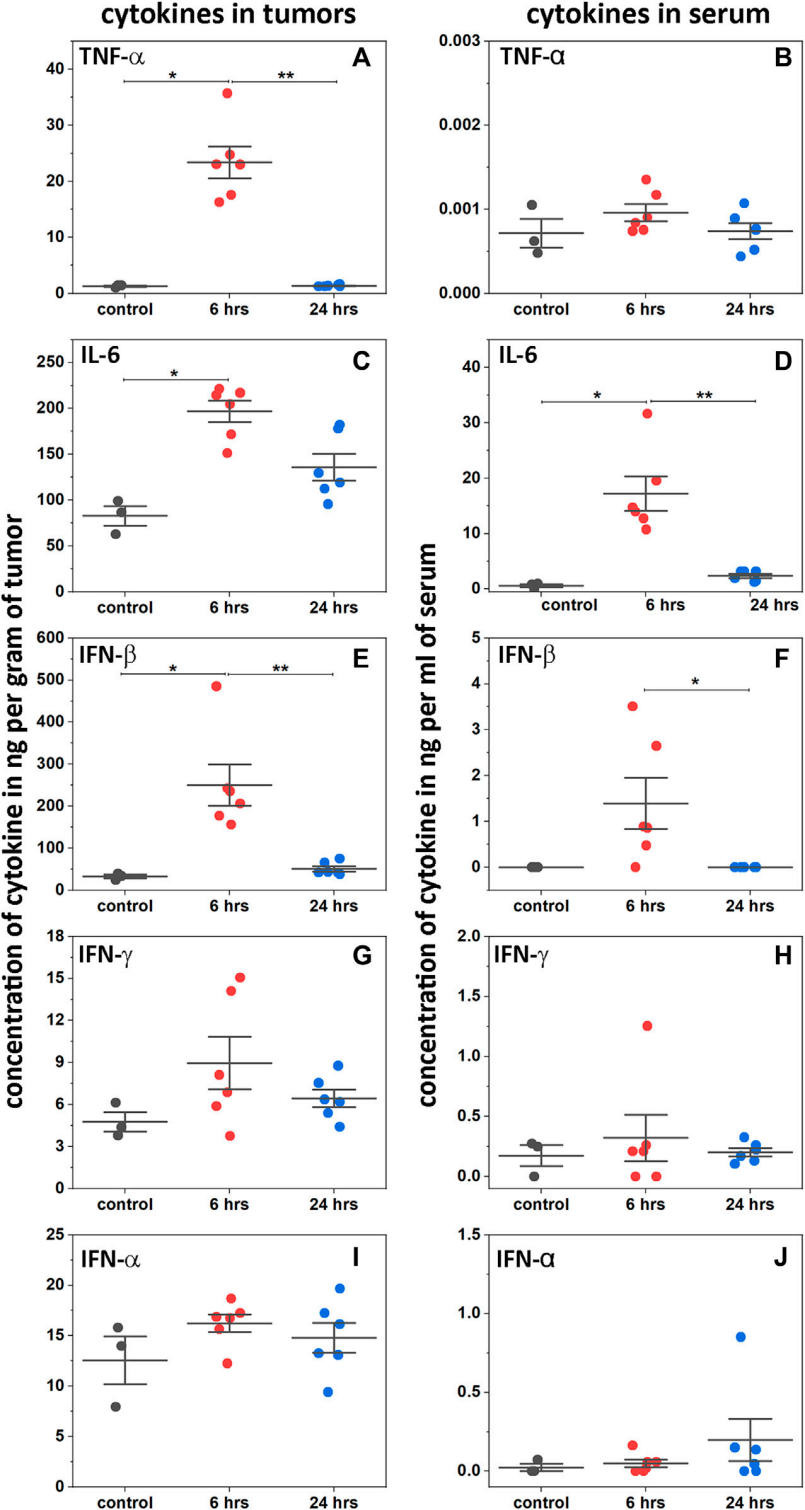
Activation of cytokines. Levels of TNF-α **(A and B)**, IL-6 **(C and D)**, INF-β **(E and F)**, IFN-γ **(G and H)** and INF-α **(I and J)** cytokines are shown in tumors and serum as established by ELISA at 6 and 24 h after a single RO injection of 2pHLIP-dMSA (300 μM 500 µL) in comparison to control mice (all points, mean, and SE are shown, * indicates that the *p*-level is < 0.02 and ** indicates that the *p*-level is < 0.002, otherwise differences are statistically insignificant, *p*-levels were calculated using the Kolmogorov-Smirnov two-tailed nonparametric test).

Activation of interferon types I and II, and induction of proinflammatory cytokines in tumors and serum were measured following a single IP injection of 2pHLIP-dMSA (300 μM 500 µL) (Figure 4). In tumors, the level of TNF-α increased 23-fold 6-h post-injection and then fell to a normal level at 24 h (Figure 4A). In serum, the level of TNF-α was insignificant both in treated and in untreated mice (Figure 4B). The blood level of IL-6 rose about 2.4-fold at 6-h post-treatment (Figure 4C) with a slight increase of its level in blood (Figure 4D), which returned to its normal level at 24 h. The level of IL-6 in serum was an order of magnitude lower compared to its level in tumors. The tumor levels of type I interferon, IFN-β, increased 8-fold in treated animals (Figure 4E), accompanied by a slight increase in serum (two orders of magnitude lower than the level in tumors) (Figure 4F). The levels of IFN-α and IFN-γ did not change much (Figures 4G–J). Thus, 2pHLIP-dMSA induced transient tumor-targeted increases of type I interferon, IFN-β, and the pro-inflammatory cytokines TNF-α and IL-6, indicating activation of the NF-kB and IRF3 signaling pathways, without systemic activation. Activation of type II interferons was not noted. The relatively fast (6 h after 2pHLIP-dMSA administration) activation of cytokines followed by their decay does not match the observed slow blood clearance. Therefore, we assessed the stability of 2pHLIP-dMSA and dMSA alone in mouse and human plasma (pHLIP peptides, especially consisting of D amino acids, exhibit high stability in plasma). The stability of 2pHLIP-dMSA (and pHLIP-mMSA) in human plasma was very high, however the stability of the agents in mouse plasma was low. The cargoes, dMSA or mMSA, were released from the pHLIP and underwent degradation within 24 h in mouse plasma. It is likely that only a fraction of injected 2pHLIP-dMSA reaches the tumor in its native form in mice.

### 3.8 Therapeutic efficacy

**FIGURE 5:**
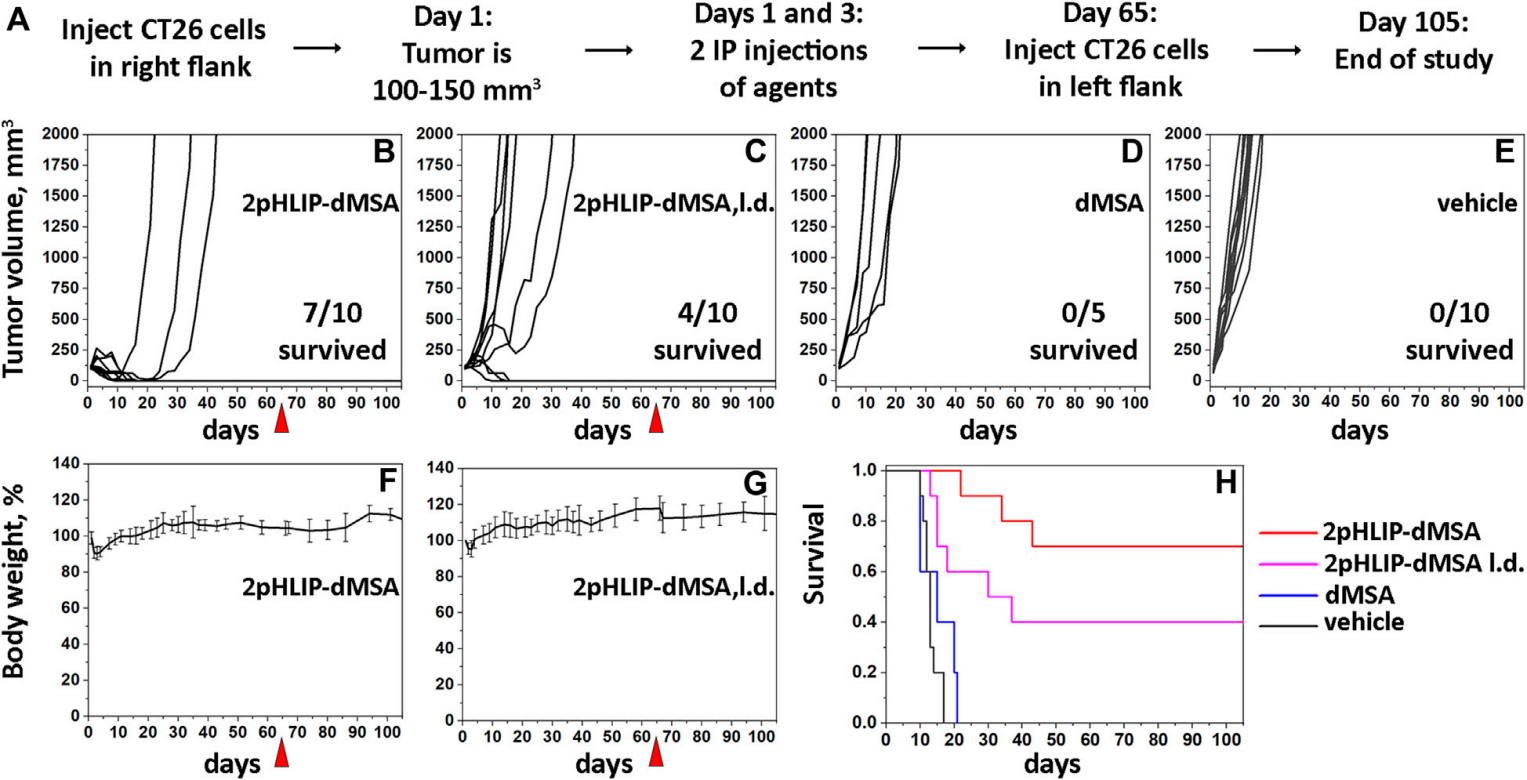
Eradication of CT26 tumors and development of immune memory. The experimental design is presented **(A)**. Right flank CT26 tumor growth curves in Balb/c mice are shown after two IP injections of 2pHLIP-dMSA **(B)**, dMSA **(D)** at dose 300 μM 500 μL per injection, 2pHLIP-dMSA at lower dose (L.d.) 100 μM 500 μL per injection **(C)**, and vehicle **(E)** performed on days 1 and 3, when tumors had reached about 100–150 mm^3^ in volume. The surviving mice were re-injected with CT26 cancer cells in the contralateral left flank on day 65, indicated by red triangle. The average changes of mouse body weights after two IP injections of 2pHLIP-dMSA **(F)** and 2pHLIP-dMSA at lower dose **(G)** are shown. Kaplan-Meier survival plots are shown **(H)**.

Due to the rapid degradation of the agent in mouse blood, we tested the therapeutic efficacy of 2pHLIP-dMSA by performing 2 injections of two different dose levels in Balb/c female mice bearing right flank CT26 tumors. When a tumor reached about 100–150 mm^3^ in volume, designated as day 1, two IP injections of 2pHLIP-dMSA, either 300 μM in 500 µL or 100 μM in 500 µL (called lower dose or l.d.) per injection, were performed on days 1 and 3 (Figures 5B,C). Tumor growth was monitored for 65 days or until the tumor volume endpoint was achieved (2,000 mm^3^, when animals were euthanized). All animals in the control groups that received either dMSA alone at a dose level of 300 μM 500 µL per injection (Figure 5D) or vehicle (Figure 5E) developed endpoint size tumors within 14–20 days p.i. In the treated groups, the tumors disappeared in 7 mice out of 10 for the higher dose and for mice treated with the lower dose of 2pHLIP-dMSA 4 out of 10 survived. 11 survivors were re-injected with cancer cells on day 65, and none of them developed tumors, indicating the development of robust immune memory. Treatment of mice led to temporary weight loss followed by complete recovery and regain of weight (Figures 5F,G). The Kaplan-Meier survival plots demonstrate dramatic differences between the controls and 2pHLIP-dMSA treated groups (Figure 5H). Thus, while dimeric STINGa, dMSA, does not exhibit any therapeutic activity by itself, when it is conjugated with two pHLIP peptides that target dMSA to the TME, the tumor is eradicated, and immune memory is developed. We also checked activity of pHLIP-mMSA at the same dose level. While mMSA did not demonstrate any activity on animals, tumor was eradicated in 2 mice out of 10 treated with pHLIP-mMSA (Supplementary Figure S9).

## 4 Discussion

We have previously tested the idea of using several pHLIPs in bundles for the targeted intracellular delivery of several cargoes. Two and four individual pHLIP peptides were bundled together by using 2-armed and 4-armed PEG polymers attached to the membrane non-inserting ends of the pHLIP peptides, while cargoes were conjugated to the membrane inserting ends of each pHLIP peptide (Wyatt et al., 2018b). This strategy gave agents containing *n* pHLIP peptides with *n* payloads, where *n* was 2 or 4. Bundling of the pHLIP peptides resulted in more efficient intracellular delivery of the cargoes. This led to the idea that multiple pHLIPs might combine their insertion energies to facilitate delivery of a single, otherwise challenging cargo.

In this study we have developed a delivery approach that uses multiple pHLIP peptides that collaborate in the targeted intracellular delivery of a single payload. Specifically, we employed two pHLIP peptides symmetrically linked to the opposite ends of a dimeric STINGa to construct the 2pHLIP-dMSA agent. We demonstrated that two pHLIPs have a kinetic advantage in translating cargo across membrane at mildly acidic pH found at the surface of metabolically active acidic cells. It was shown previously that pHLIP can extend blood circulation of small molecules by an order of magnitude (Crawford et al., 2020; Gayle et al., 2021; Moshnikova et al., 2022b). Notably, 2pHLIP-dMSA exhibits the longest circulation in blood (half-life >30 h) among other investigated pHLIP-cargo conjugates. As a result, blood and tissue clearance was slow, while tumor targeting was significant. Importantly, activation of type I interferon and pro-inflammatory cytokines were high and transient in tumors without systemic activation, which is crucial for the development of safe and effective STINGa treatments. Tumors were eradicated in seven out of ten mice treated with 2pHLIP-dMSA. In the other three animals, the tumor initially disappeared, however later they redeveloped the tumors, which quickly progressed to large sizes. In the group treated with the lower dose of 2pHLIP-dMSA, just 4 animals out of 10 survived. Others either developed tumors quickly or with some delay. All survived animals, irrespective of dose of treatment, did not develop tumors after re-challenge with another injection of cancer cells. At the same time non-targeted dMSA at dose equivalent to the highest dose used in the experiment, was non-effective in treatment of tumors. It is interesting to note that therapeutic effect of targeted STINGa is threshold-based. Either the immune system is actively reacting to the treatment, which leads to the tumor eradication and development of a very robust protective response, or there is an escape and tumor will develop quickly. At the higher dose of treatment, the probability of escape is lower, while at the lower dose of treatment the probability of escape is increasing. At the same time, the responded animals have similar effects in groups of higher or lower doses. More studies would be required to understand which parameters drive an escape. Potential successful treatment should be associated with strong, but transient effect without systemic activation, which could be achieved by targeted delivery of STINGa to immune, tumor stroma and cancer cells.

Our results demonstrate that the energy of pH-triggered membrane-associated folding of two pHLIP peptides could be utilized in targeted intracellular delivery of a payload. It could be especially advantageous for intracellular delivery of large, potentially polar, and challenging therapeutic payloads like dimers of STINGa, peptide nucleic acids or other therapeutic cargo molecules.

The STING pathway plays a central role in regulating the anti-tumor immune response. However, untargeted use of STINGa is associated with serious side effects and the clinical utility is poor. Thus, there is a significant effort underway to introduce and develop targeted approaches for more effective delivery of STINGa. Immune-stimulating antibody-conjugates (ISACs) are under development and have entered clinical trials. An antibody against, the epidermal growth factor receptor (EGFR) conjugated with STINGa via cleavable linker, demonstrated enhanced preclinical efficacy (Wu et al., 2022). STINGa conjugated with human epidermal growth factor receptor 2 (HER2), Mersana’s XMT-2056 conjugate, has demonstrated very good preclinical results as well. However, a grade 5 (fatal) serious adverse event was observed during clinical trials. Takeda undertook a different approach by developing TAK-500, an IgG1 anti-cysteine-cysteine chemokine receptor 2 (CCR2) antibody conjugated with their TAK-676 STINGa via a self-immolating maleimide-containing protease-cleavable peptide linker, targeting TAMs. Currently TAK-500 is in clinical trials and is showing good activity (Diamond et al., 2022). Another approach is to use nanomaterials for the encapsulation and protection of STINGa. Lipid nanodiscs (LNDs), formed from the self-assembly of PEGylated lipids encapsulated with cyclic dinucleotide (CDN) were successfully tested in preclinical tumor models (Dane et al., 2022). Nanoparticles comprising a pH-sensitive polymer with a cyclic seven-membered ring (PC7A) and multiple STINGa molecules were also tested preclinically and showed improved performance compared with use of STINGa alone (Li et al., 2021).

pHLIP has several potential advantages compared with other delivery mechanisms: 1) pHLIP senses and targets pH at the surface of all acidic cells within the TME including cancer cells, TAMs, CAFs, mMDSCs, and DCs in a manner that is not dependent upon molecular tumor biomarkers, which change and can be evaded as a result of ongoing selection pressure, 2) pHLIP efficiently translocates large polar molecules directly into the cytoplasms of acidic cells; and 3) pHLIP exhibits improved tumor penetration compared with antibodies and nanoparticles. Our previous and current studies demonstrate significant promise in use of pHLIP for targeted intracellular delivery of dimeric STINGa. Specifically, employing the combined energy of the membrane-associated folding of two pHLIP peptides for the delivery of a single dimeric STINGa offers a number of advantages including a kinetic advantage in the translocation of cargo across the membrane, long blood circulation, excellent tumor targeting and selective activation of cytokines in the TME without systemic activation. The prolonged, 1–2 days, circulation of the 2pHLIP-dMSA in the blood leads to the improvement of tumor targeting and delivery of sufficient amounts of STINGa to tumors, which is required to induce strong transient activation of the STING pathway in cancer, stromal and immune cells simultaneously. At the same time, prolonged circulation increases the requirement for stability of the agent in the plasma. While the agent exhibits better stability in human plasma compared to mouse plasma, searching for more stable agents, with better linkers connecting the pHLIP peptides to dimeric STINGa, will be desirable. Moreover, the kinetics of self-immolation were slow and complex, which would require improvement prior to clinical translation.

Besides the challenge of the targeted delivery of STINGa, there is also a biological limitation associated with the silencing of the STING pathway in variety of tumors (Konno et al., 2018). In a TCGA based analysis it was reported that STING expression is lost or downregulated mainly through promoter hypermethylation-driven silencing, and silencing can be restored by employing DNA methylation inhibitors (Konno et al., 2018; Falahat et al., 2019; Falahat et al., 2021; Low et al., 2022; Falahat et al., 2023).

Combining approaches for targeting of STINGa and restoring STING expression by DNA methylation inhibitors, when needed, may lead to a breakthrough in clinically effective STING-therapies.

## Data Availability

The original contributions presented in the study are included in the article/Supplementary Material, further inquiries can be directed to the corresponding author.
