# Supplementary material for: Targeted intracellular delivery of dimeric STINGa by two pHLIP peptides for treatment of solid tumors
Source: Front Pharmacol. 2024 Mar 1;15:1346756. doi: 10.3389/fphar.2024.1346756 (PMC10940318; doi:10.3389/fphar.2024.1346756)
Supplement: Supplementary file 1 [file DataSheet1.docx]

**Supplementary Information**

**Targeted intracellular delivery of dimeric STINGa by two pHLIP peptides for treatment of solid tumors**

**Table S1. Competitive binding assay.** DNSA and DNSG fluorescent dye binding constants with HSA (*K*_DNSA_ and *K*_DNSG_) in the absence and presence of pHLIP, pHLIP-mMSA, 2pHLIP-dMSA, warfarin and ibuprophen.

| **Agent, concentration** | | ***K*_DNSA_,** µM | ***K*_DNSG_,** µM |
| --- | --- | --- | --- |
| **pHLIP,** | 0  5 µM  10 µM | 22.7 ± 1.3  52.2 ± 0.3  48.6 ± 0.8 | 6.2 ± 0.6  10.9 ± 0.9  15.6 ± 1.3 |
| **pHLIP-mMSA,** | 0  5 µM  10 µM | 22.7 ± 1.3  29.2 ± 1.6  32.0 ± 1.7 | 6.2 ± 0.6  23.4 ± 1.3  30.2 ± 1.6 |
| **2pHLIP-dMSA,** | 0  5 µM  10 µM | 22.7 ± 1.3  47.7 ± 4.4  53.1 ± 4.2 | 6.2 ± 0.6  48.6 ± 4.9  24.0 ± 1.8 |
| **warfarin,** | 0  5 µM  10 µM | 22.7 ± 1.3  58.4 ± 4.6  120.6 ± 11.0 | - |
| **ibuprofen** | 0  5 µM  10 µM | - | 6.2 ± 0.6  32.1 ± 2.7  32.3 ± 2.7 |

**
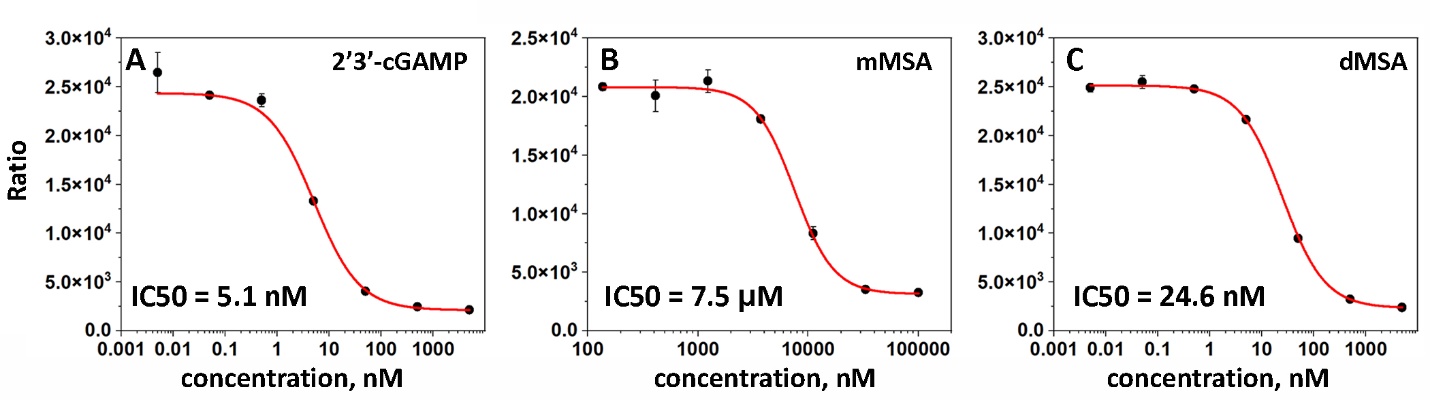
**

**Figure S1. Construct binding to STING protein**. Binding of 2’3’-cGAMP (control) (A), monomeric mMSA (B) and dimeric dMSA (C) with human STING WT protein was measured by the HTRF binding competitive assay, which uses d2-labeled STING ligand, a 6His tagged human STING protein, and an anti 6His cryptate-labeled antibody. Investigated STINGa were competing with the STING ligand-d2 and prevented FRET. IC50 values are from curve fitting (red).


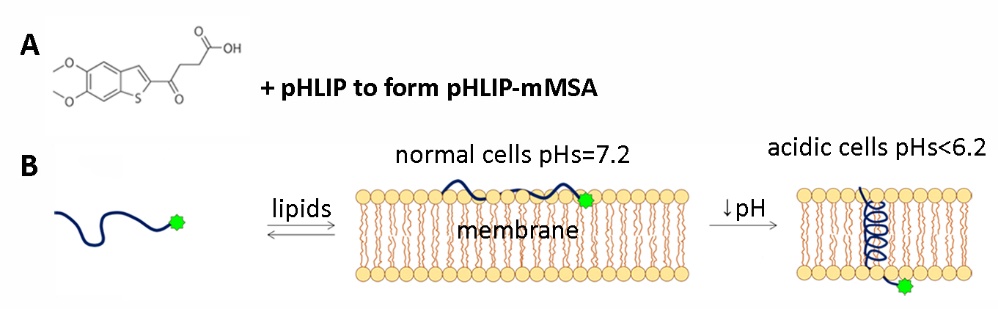


**Figure S2. Schematic representation of pHLIP-mMSA interaction with a membrane**. (A) The chemical structure of mMSA. (B) shows schematics of pHLIP-mMSA interactions with a cell membrane with normal surface pH (pH =7.2) in healthy tissues and membrane of acidic cells with surface pH <6.2 found in diseased tissues. The green circle represents monomeric MSA.


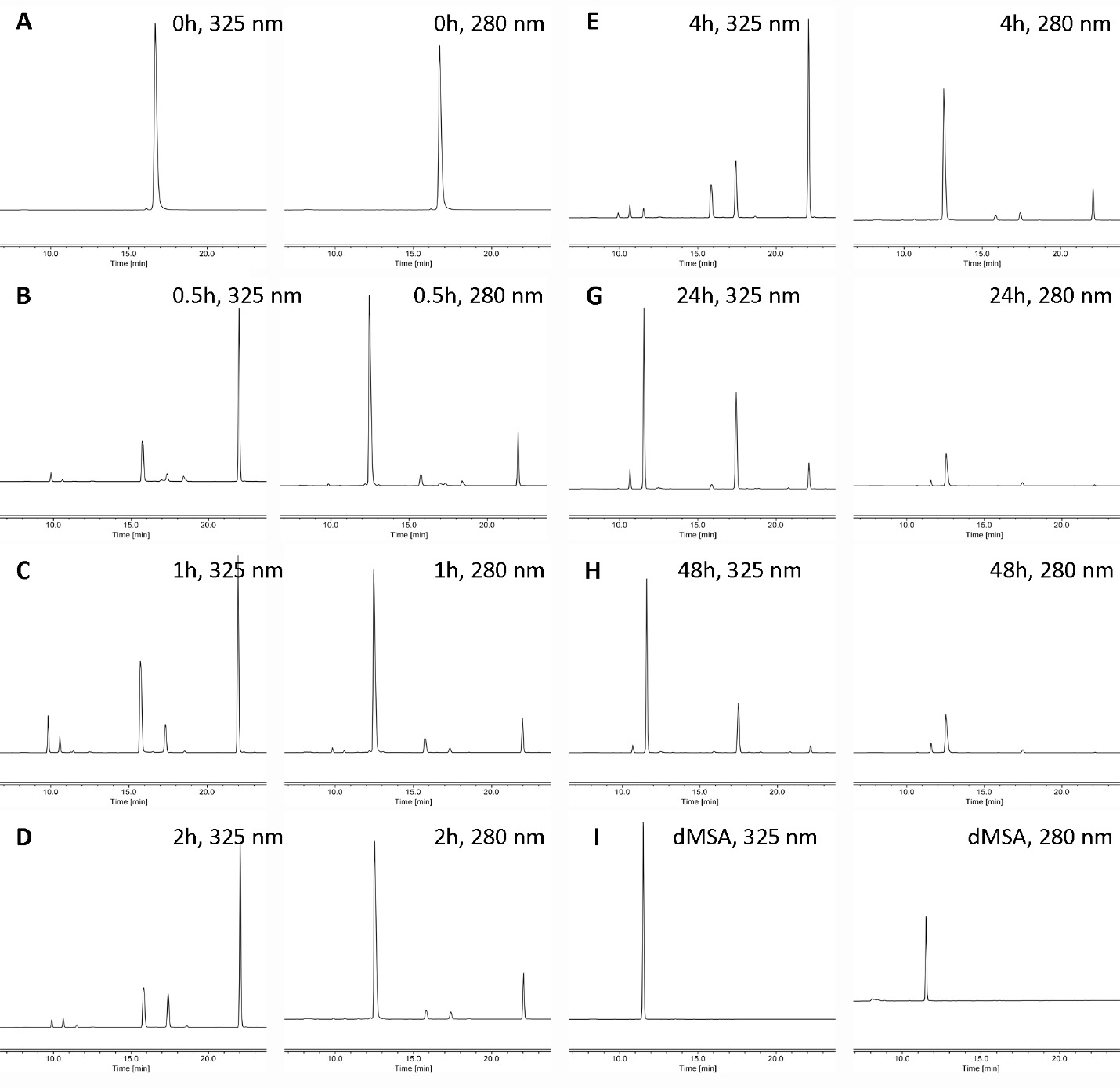


**Figure S3. Kinetics of self-immolation of 2pHLIP-dMSA**. HPLC chromatograms recorded at 280 nm and 325 nm for 2pHLIP-dMSA treated with DTT dissolved in 50 mM phosphate buffer containing 150 mM NaCl pH 7.4 at different time points (0, 0.5, 1, 2, 4, 24, 48 hrs) after addition of DTT (B-H), and the retention time for dMSA (I) are shown. Starting 2pHLIP-dMSA is shown on A.

**
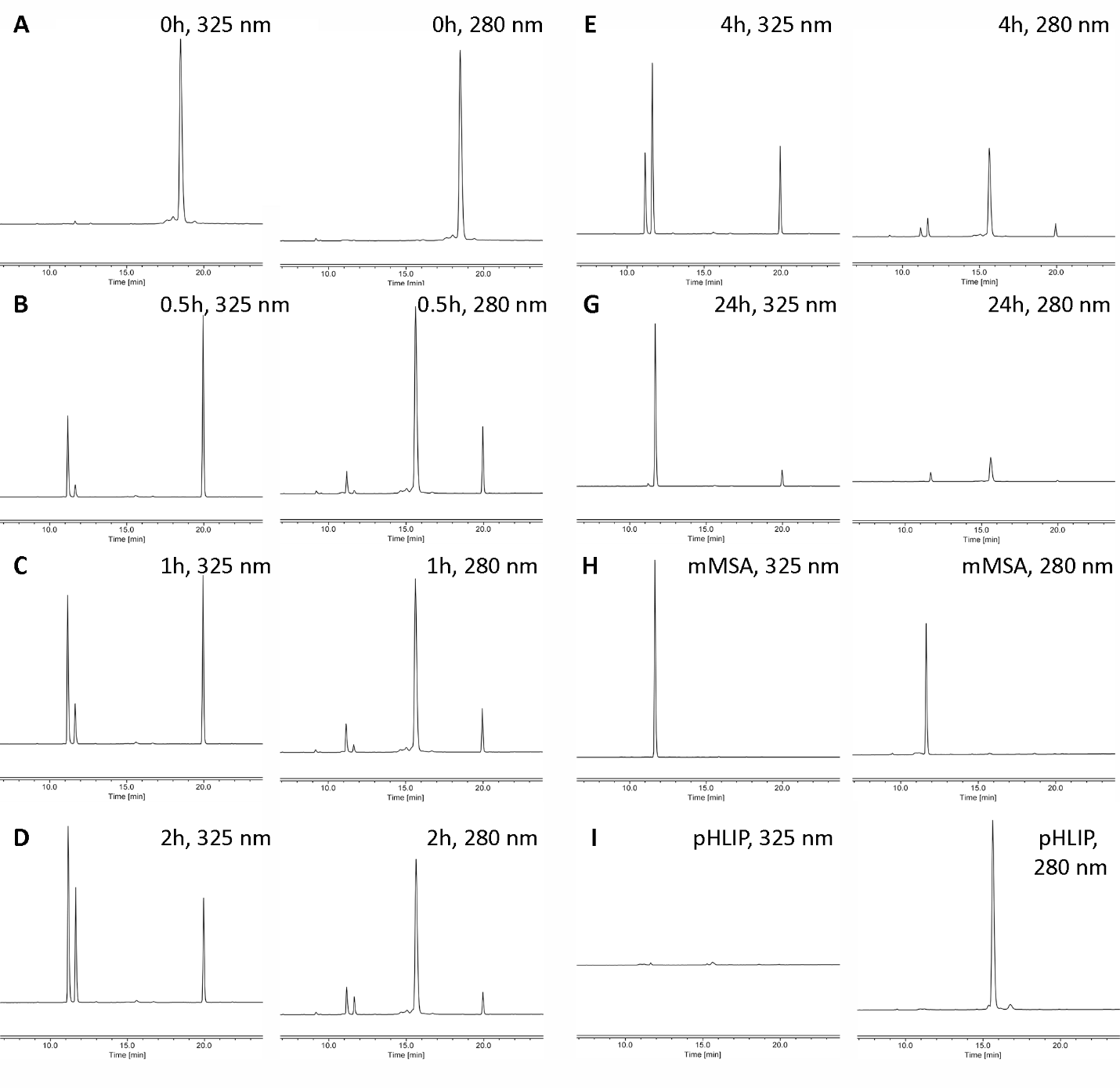
**

**Figure S4. Self-immolation kinetics of pHLIP-mMSA**. HPLC chromatograms recorded at 280 nm and 325 nm for pHLIP-mMSA treated with DTT dissolved in 50 mM phosphate buffer containing 150 mM NaCl pH 7.4 at different time points (0, 0.5, 1, 2, 4, 24 hrs) after addition of DTT (A-G) and the retention time for mMSA (H) and pHLIP (I) are shown. Starting pHLIP-mMSA is shown on A.


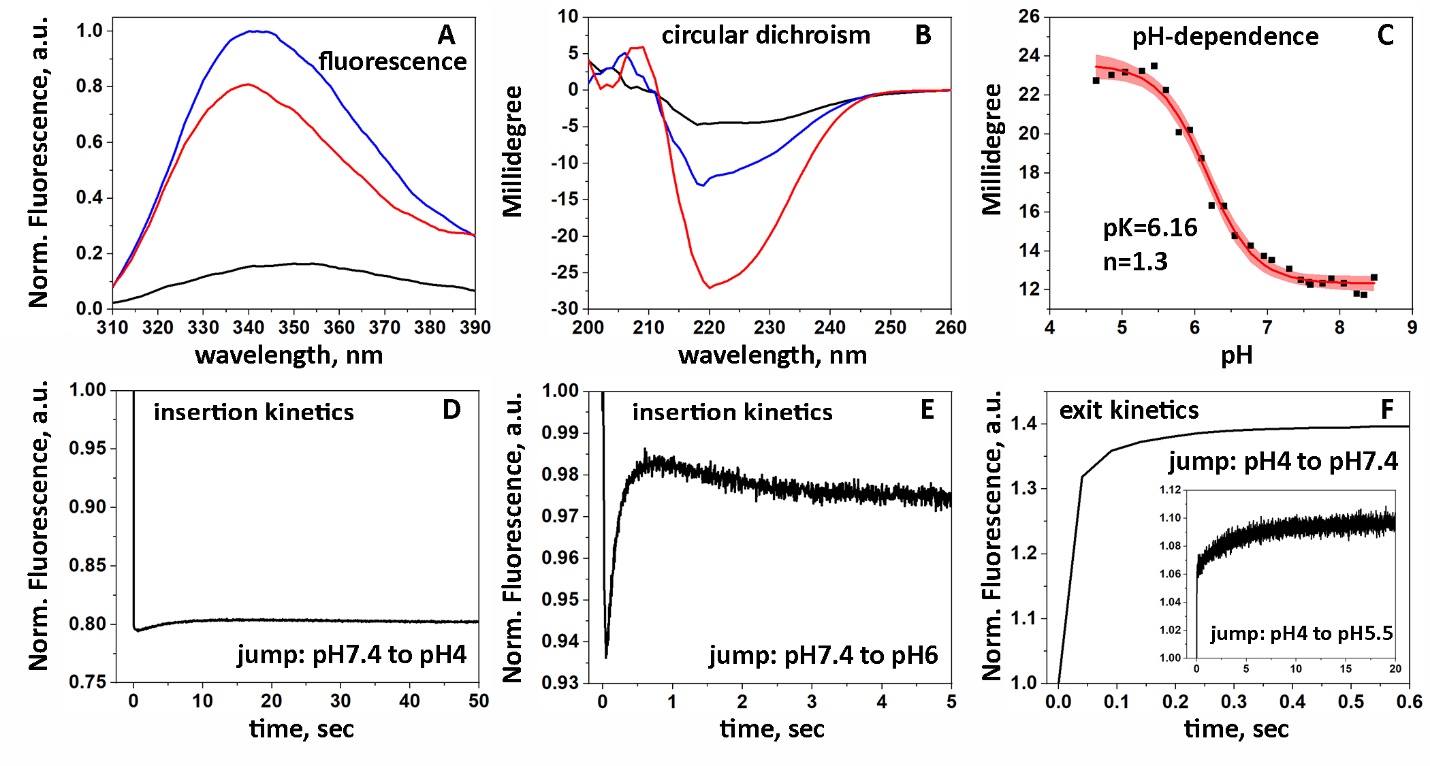


**Figure S5. Interaction of pHLIP-mMSA with liposome lipid bilayers.** Fluorescence (A) and CD (B) spectra of pHLIP-mMSA in solution at pH 7.4 (black lines), in the presence of POPC liposomes at pH 7.4 (blue lines) and in the presence of POPC liposomes at pH 3.5 (red lines) are shown. CD spectral signals were multiplied by (-1) since D-amino acid pHLIP peptides were used in the study. pH-dependent transitions monitored by changes of the CD spectral signal in the presence of POPC liposomes (experimental points and fitting curves, red, with 95% confidence interval, pink) is shown (C). Kinetics of fluorescence changes triggered by a pH drop (D, E) or pH increase (F) in the presence of POPC liposomes are shown.

**
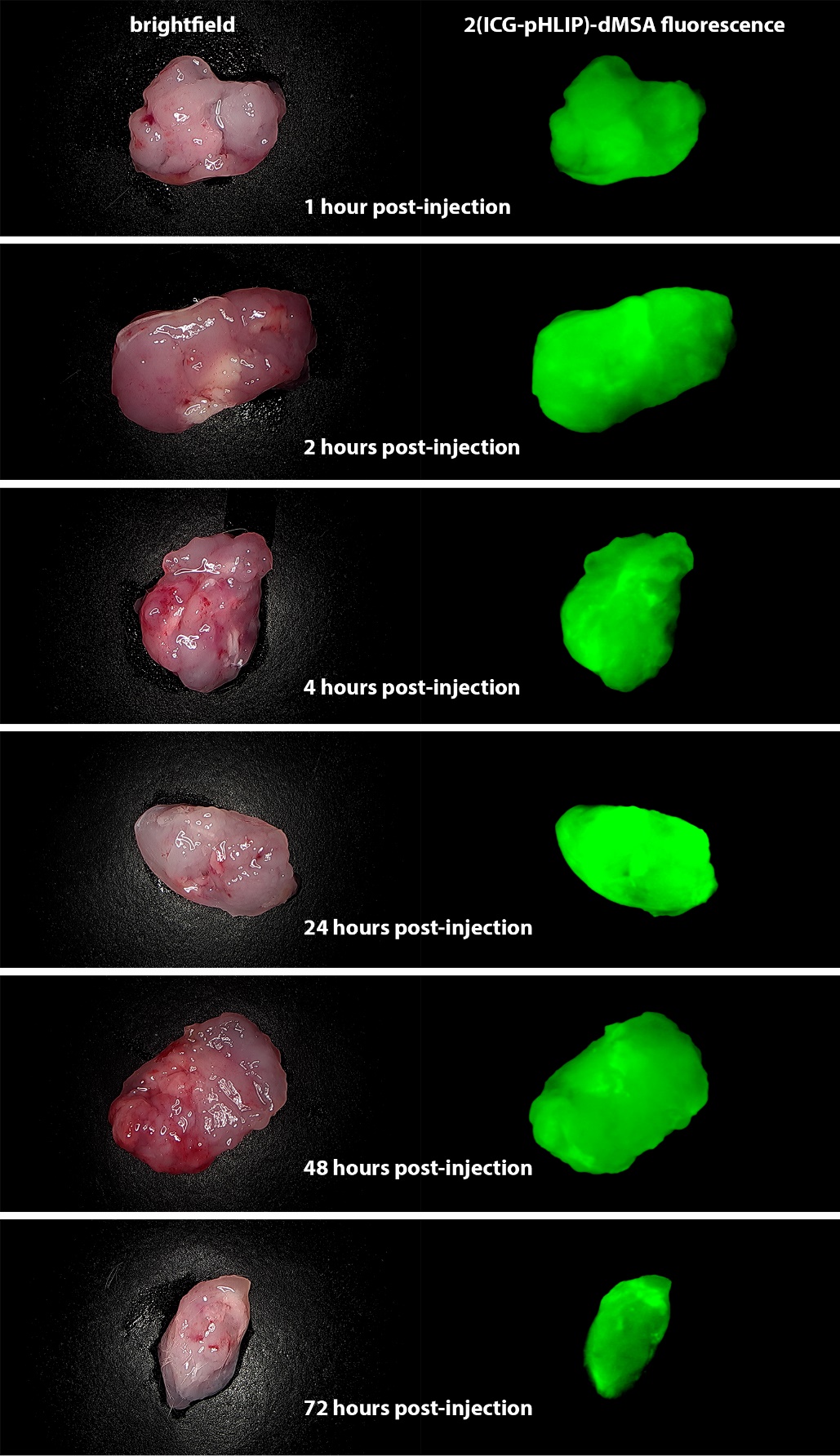
**

**Figure S6. Imaging of 2(ICG-pHLIP)-dMSA in tumors.** Representative images of tumor pieces recorded at selected time points after a single RO injection of 2(ICG-pHLIP)-dMSA. Animals were euthanized at each time point, and tumors were collected and imaged. Brightfield and fluorescent images (green) are shown.


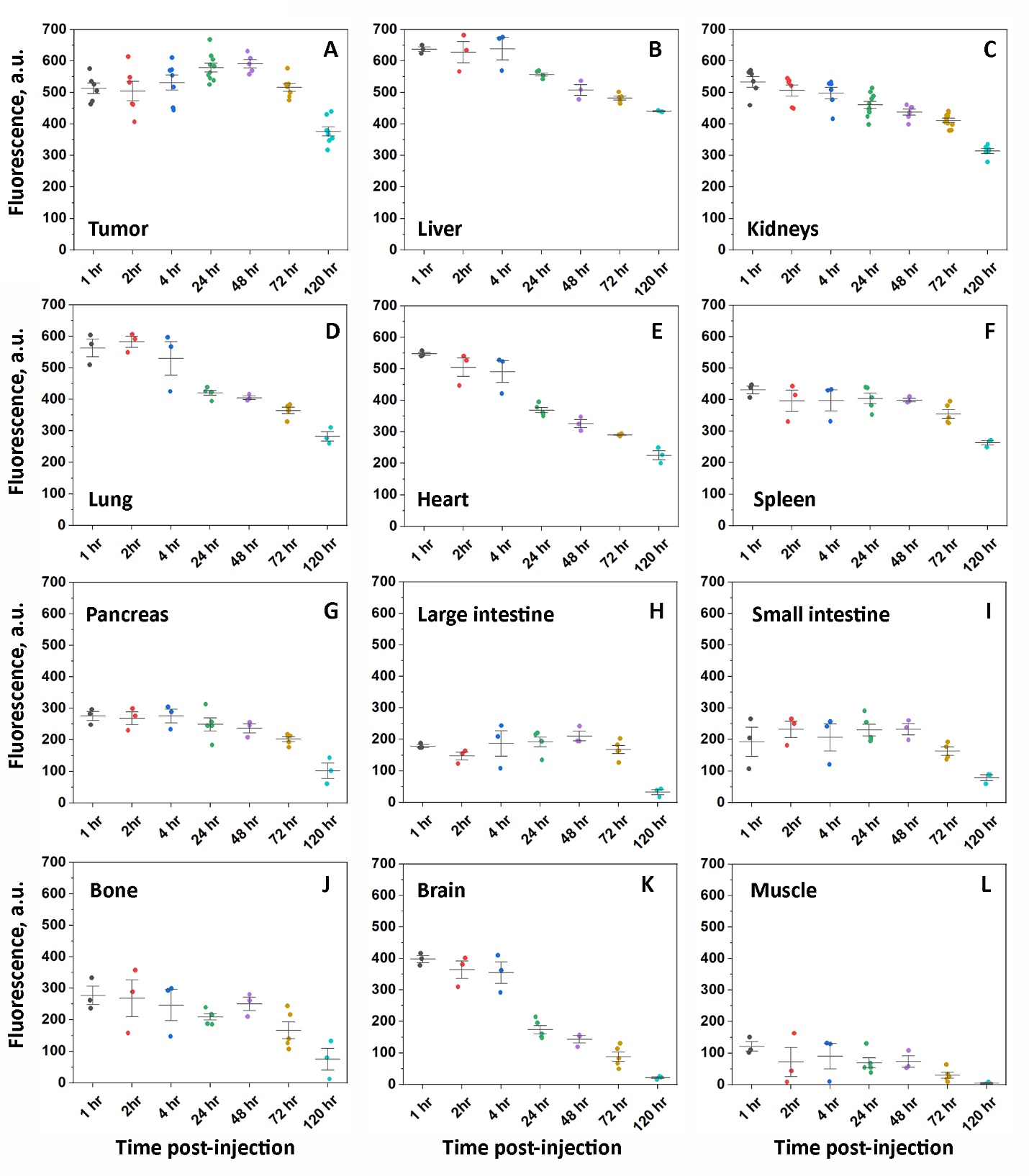


**Figure S7. Biodistribution of 2(ICG-pHLIP)-dMSA in organs and tissues.** Mean fluorescence per area was calculated for each tissue and organ at different time points after a single RO injection of the agent. All points, means, and standard errors are shown.

**
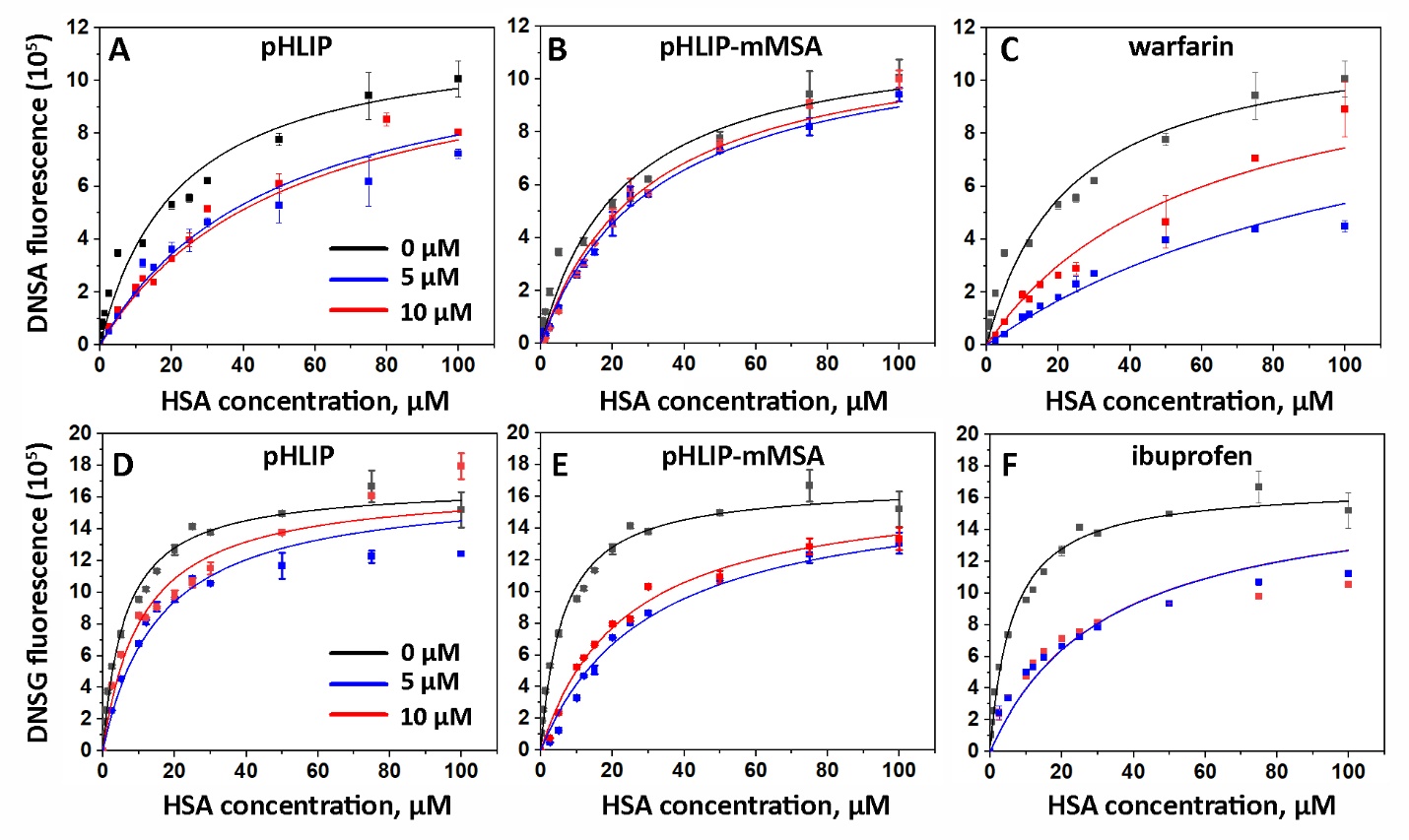
**

**Figure S8. HSA binding.** Changes of DNSA (A-C) and DNSG (D-F) fluorescence with increasing HSA concentration in absence (black symbols) or presence of 5 µM (blue symbols) or 10 µM (red symbols) of pHLIP (A and D), pHLIP-mMSA (B and E), warfarin (C) and ibuprofen (F). The global fitting was performed with each set of data and the fitting curves are shown as black, blue and red lines.


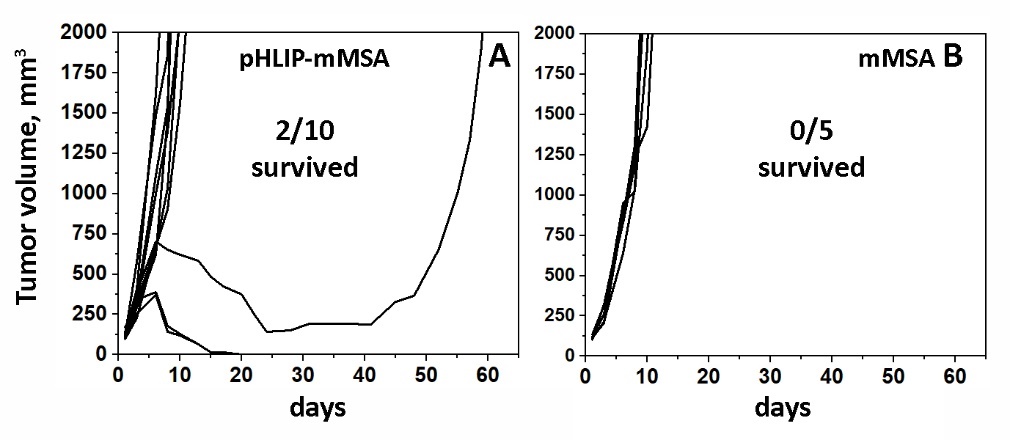


**Figure S9. Treatment with pHLIP-mMSA and mMSA.** Right flank CT26 tumor growth curves in Balb/c mice are shown after two IP injections of pHLIP-mMSA (A) and mMSA (B) at dose 300 μM 500 μl per injection performed on days 1 and 3, when tumors had reached about 100-150 mm^3^ in volume (designated as day 1).
